# Inequality in learning is a major concern after school closures

**DOI:** 10.1073/pnas.2105243118

**Published:** 2021-04-21

**Authors:** Herman G. van de Werfhorst

**Affiliations:** ^a^Department of Sociology, University of Amsterdam, Amsterdam 1001 NA, The Netherlands;; ^b^Amsterdam Centre for Inequality Studies (AMCIS), University of Amsterdam, Amsterdam 1001 NA, The Netherlands

The COVID-19 pandemic has come with many nonpharmaceutical interventions to curb the spread of the virus. One of the most significant measures has been to close schools for in-school education, at the primary, secondary, and tertiary levels of schooling. While such interventions have been demonstrated to be effective in reducing the spread of the virus (1), they come at a cost: an intermittence of the learning process that is not easily repaired with online instruction. The well-designed study by Engzell et al. (2) shows, convincingly, that the “learning loss” due to school closures is severe (although learning delay may be a more appropriate term). Using data from primary schools in The Netherlands and analyzing test scores on externally standardized tests, the study reports a learning loss of 3.16 percentiles on a composite index of math, reading, and spelling, an effect that varies in size by socioeconomic background and school composition.

At least as concerning as the overall learning delay is the reported inequality by socioeconomic background and school composition. Using school records of parental educational attainment (classifications used to determine weighted student funding for schools with children from disadvantaged backgrounds), the study shows that children of very low-educated parents (i.e., none of the parents have more than lower-secondary education; in total, 8% of the families) suffer more from school closure than children from more-educated backgrounds. Using a more fine-grained measure of school-level disadvantage, supplementary analyses show that the learning delay is much stronger in schools with a higher share of disadvantaged children (which could be simply compositional, as the school-level indicators were not available at the individual student level). Other European studies on the impact of COVID-19−related school closures show similar patterns (3, 4), with stronger delays among children with disadvantaged backgrounds or schools with higher concentrations of disadvantage.

Possible explanations for a learning delay gap are related to parental involvement with education (5), socioeconomic differences in information and communications technology (ICT) access and skills among students and the schools they attend (6, 7), and parents’ ability to help with homework during the school closures (8). More studies are needed to understand these mechanisms. Following the “digital divide” literature, providing students with technological devices alone is unlikely to solve the problem, as inequality in digital skills and usage are additional sources of the digital gap between socioeconomic groups (9).

In interpreting effect sizes, Engzell et al. (2) estimate that the average learning progress has been close to zero during the closures. The overall learning delay of 3.16 percentiles equals 0.08 SDs. Comparing this to the estimated SD growth from American studies and the World Bank of 0.40 SD learning growth per year under normal circumstances, the study concludes that the loss equals 8 wk of lost progress, similar to the 8-wk school closure (close to one-fifth of a school year). If, instead of using American or global estimates on school progress under normal circumstances, we use Dutch estimates on the spelling test that is analyzed [available for spelling growth between Dutch grades 6 and 7, roughly from age 10 y to 11 y (10)], we can further benchmark the estimated 3.03 percentile learning loss in spelling for this grade, in SI Appendix, table S11 of ref. 2. The national SD growth equals 0.64 SD growth on the midyear test and 0.45 SD at the end-of-year tests between grades 6 and 7, averaging 0.55 SD (calculations based on table 2.1 of ref. 10). Assuming 3.03 percentile loss equals (3.03/3.16*0.08 =) 0.077 SD, the learning delay would then be roughly 0.077/0.55 = 0.14 of a school year, equaling 5.6 wk of learning delay over the 8-wk closure (assuming a 40-wk school year). Following these rough estimates, the closure weeks then worked at a 30% efficiency. The study by Engzell et al. (2) goes a long way in interpreting the effect sizes, and they too present some estimates that suggest there may be some, but strongly reduced, efficiency of the online learning process that replaced in-school instruction.

Two control variables have been inserted in the difference-in-differences models of ref. 2: an overall trend over years and the number of days between the midyear and end-of-year tests. Without these control variables, the negative impact of school closures is less strong. In defense of their inclusion of the trend parameter, the placebo analysis had more expected null effects than without a trend control. The trend parameter is positive, suggesting that, across the years 2017−2020, school performance growth has increased, while other student assessment data on students in Dutch grade 6 show a stable or declining level of literacy (11) and mathematics (12). This could be due to the difference between levels and growth (i.e., lower growth at higher levels). Unfortunately, the study does not show trend parameters in the comparison years only (the placebo analysis is only reported graphically, and not in table form with all coefficients).

The control for number of days between tests requires somewhat more thought. Under normal circumstances, a larger number of days between tests comes with higher performance due to prolonged learning, but, in the treatment (pandemic) year, the second test was taken later, while performance was lower. This means that the number of days between tests functions as a suppressor variable, as the treatment year is positively associated with number of days between tests, and negatively associated with student performance, while the relationship between number of days and student performance is positive. As the leverage of the (positive) slope originates from the comparison years, a linear control for number of days for all years together is likely not a functional form that would fit the data well, but the model does not allow differential effects by treatment status. The robustness checks in ref. 2 report, indeed, that the negative effect of school closures is reduced by 12% if the control variable is omitted.

The study of Engzell et al. (2) demonstrates how much can be learned about student progression during the pandemic if the data infrastructure is up to the task. It uses well-known externally validated and standardized tests, made available to the researchers through an alliance with a provider of analytics services to schools. Recently, other data on the same and other student assessments have become available for researchers. Building upon new secure register-based school career data of the National Cohort Study on Education (Nationaal Cohortonderzoek Onderwijs) (13), student monitoring data can be connected to further school careers, and student socioeconomic and migration background, in more detail than in ref. 2. Recent reports on the effects of the school closure using these data show comparable results (14): a decline of between 14% (spelling) and 25% (reading) relative to prepandemic years, and strong socioeconomic gaps in the decline also in more fine-grained measures. Gaps by migration background (net of socioeconomic background) are smaller but found for reading.

**Fig. 1.**
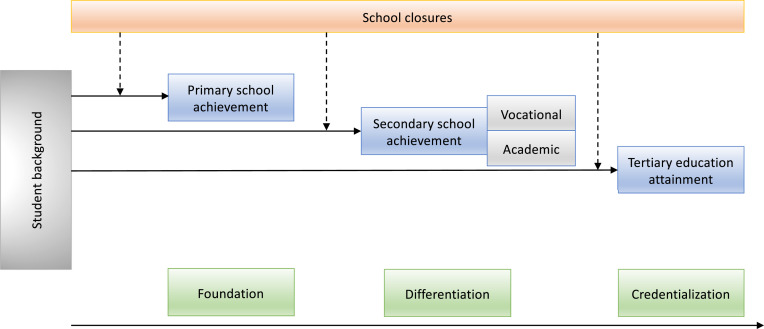
Possible school closure effects for the educational career.

Importantly, the rich Dutch data infrastructure also enables researchers to closely monitor student progression in the years to come. To help students get back on track, schools also can be informed regularly about their students’ performance in comparison to schools that have a wide range of similar characteristics. Moreover, researchers can study school careers beyond primary and secondary education. This is relevant, as the effects of school closures may be different across the school career. From a sociological perspective, primary education is mostly concentrated on the foundation of basic skills, secondary education is mostly concentrated on differentiation in learning trajectories, and tertiary education is mostly focused on credentialization: acquiring qualifications that provide access to the labor market (Fig. 1). Impacts of school closures on inequalities in learning (the dotted arrows) may be most prominent in primary education, because the foundation is laid for future learning progression. Closures can furthermore impact socioeconomic inequalities in differentiation in secondary education, especially when the transition from primary to secondary education happens after periods of closure, or when standardized tests have been cancelled during school closures (15). Swiss research showed that online instruction during school closures was equally as effective as in-school teaching at the secondary level, while primary education was more negatively affected by online instruction (16). Inequalities in tertiary education may also be less strongly affected by closures, as the credentialization process continues. A study on a 1968 French protest, which led students to be given their upper-secondary baccalauréat diploma easily, showed that these students have not suffered from it. More of them graduated from university, and they received higher earnings compared to a counterfactual when the diploma had been as selectively awarded as before (17).

Governments and schools face a heavy task: How can the delay in learning be remedied? Some governments are willing to spend substantial funds to this challenge. The Dutch government has, for instance, allocated EUR 8.5 billion over 2.5 y, or 3.4 billion per year, which equals around 8% of the yearly budget for the Ministry of Education, Culture and Sciences. A high-quality data infrastructure can help the field to understand the effectiveness of promising (inequality-reducing) interventions such as offering after-school tutoring (18), enrolling students in summer programs (19), and employing additional teachers and teacher assistants (20), provided that such interventions are focused and intensive as these are known conditions for success. Countries across the globe can benefit from the lessons learned from such interventions, provided that a solid research infrastructure exists to monitor their effects.
